# Direct observation of single-atom defects in monolayer two-dimensional materials by using electron ptychography at 200 kV acceleration voltage

**DOI:** 10.1038/s41598-023-50784-z

**Published:** 2024-01-02

**Authors:** Ying Chen, Tzu-Chieh Chou, Ching-Hsing Fang, Cheng-Yi Lu, Chien-Nan Hsiao, Wei-Ting Hsu, Chien-Chun Chen

**Affiliations:** 1 Department of Engineering and System Science, National Tsing Hua University, Hsinchu, 300044 Taiwan; 2 Department of Physics, National Tsing Hua University, Hsinchu, 300044 Taiwan; 3 Taiwan Instrument Research Institute, National Applied Research Laboratories, Hsinchu, 300092 Taiwan

**Keywords:** Materials science, Nanoscience and technology

## Abstract

Electron ptychography has emerged as a popular technology for high-resolution imaging by combining the high coherence of electron sources with the ultra-fast scanning electron coil. However, the limitations of conventional pixelated detectors, including poor dynamic range and slow data readout speeds, have posed restrictions in the past on conducting electron ptychography experiments. We used the Gatan STELA pixelated detector to capture sequential diffraction data of monolayer two-dimensional (2D) materials for ptychographic reconstruction. By using the pixelated detector and electron ptychography, we demonstrate the observation of the radiation damage at atomic resolution in Transition Metal Dichalcogenides (TMDs).

## Introduction

In 1952, Sayre^1^ applied Shannon’s sampling theory to propose a theoretical framework for crystal diffraction to reconstruct objects from diffraction patterns. In 1980, Sayre^2^ suggested the use of a higher sampling density for the reconstruction of single, isolated non-periodic samples. This approach eventually led to the first X-ray diffraction report of isolated samples in 1987^3^. Finally, the first lensless X-ray imaging technique experiment, known as Coherent Diffraction Imaging (CDI), was completed and reconstructed by Miao in 1999^4,5^. With the phase retrieval algorithm, the resolution of this lensless microscopy is no longer constrained by the size of a focused spot; instead, it is redefined by the scattering angles and wavelengths. However, CDI still needs some improvements, including its high requirement for coherence, the need for a high oversampling ratio of the detector, and constraints on the field of view limited by the incident beam size.

The X-ray ptychography^6,7^, a combination of scanning transmission X-ray microscopy (STXM) with CDI, was initially conceptualized by Hegerl and Hoppe in 1970^8^ to address phase problems in crystallography. In real space, 2D scanning generates a 2D diffraction pattern for each coordinate in reciprocal space. This results in a complete 4D data cube. Starting in 1989, Maiden and Rodenburg et al. introduced a series of advancements, including Wigner-distribution deconvolution (WDD)^9^, single-sideband (SSB)^10^, and the widely applied ptychographic iterative engine (PIE)^11^. The first experimental validation was completed in 2007^12^. Subsequently, several attachments were incorporated into ptychography. For example, the extended version (ePIE) to modify the probe and object simultaneously^13,14^, the position correction (pcPIE) with coordinate adjustments during iterations^15^, and the Mix-state^16,17^ to handle source partial coherence.

In electron ptychography, researchers such as Nellist^18^, Rodenburg^19^, Maiden^20^ et al. have made significant contributions to overcome the shortcomings of conventional lens imaging. The detector system has also undergone significant evolution, transitioning from earlier methods involving fluorescent viewing screens (powdered ZnS or ZnS/CdS) and film-based systems (comprising layers of gelatin and silver halide emulsion). In the 1980s, a more modern approach involving Charge-Coupled Device (CCD) camera-based systems^21,22^, typically coupled with a scintillator and cooling device, was introduced. As we entered the 2000s, complementary metal oxide semiconductor (CMOS) electronics became widely available for transmission electron microscopy (TEM)^23^. CMOS cameras offered faster readout speeds and minimized charge spreading from oversaturated pixels compared to CCD cameras.

To perform ptychography using the electron source, the detector needs to possess single-electron sensitivity and achieve high dynamic range efficiency over multiple orders of magnitude at high scattering angles. Additionally, it requires fast imaging capabilities to reduce the sample stage’s drifting, thermal vibration, and instability. This has also posed a challenge for electron ptychography, a subset of 4D STEM techniques, which had been suffering from inadequate hardware development of 2D detectors until the advent of direct electron detectors.

In 2016 the electron microscope pixel array detector (EMPAD) was developed^24^. Two years later, D. Muller’s group at Cornell University achieved groundbreaking electron ptychography based on the EMPAD. The reciprocal space information limit is nearly 5 $\alpha$ (107 mrad) at an acceleration voltage of 80 *kV* and achieved a resolution of around 0.39 Å^25^. In 2020, Plotkin-Swing, Benjamin et. al.^26^ demonstrated the hybrid pixel direct detector. This detector’s remarkable attributes, including sensitivity and large pixel count ideal for low-dose imaging and in-situ studies, render it highly suitable for electron energy loss spectroscopy (EELS). In 2022, another direct detector, STELA^27–29^, was produced by Gatan, Inc. with high speed and high dynamic range ideal for diffraction imaging at low kV. Those direct detectors no longer rely on phosphor conversion for light before photon counting. More specifically, they count individual electron events, resulting in counts free from readout or amplifier noise. Additionally, a hybrid pixelated detector can maintain a linear response even under high electron flux, making it more suitable for electron scattering experiments.

Here we present electron ptychography of monolayer molybdenum disulfide (MoS$_2$) using the Thermo Fisher Cs-corrected Titan equipped with the STELA pixelated detector at the Taiwan Instrument Research Institute. We also conducted scanning step tests with both focused and defocused conditions on monolayer tungsten disulfide (WS$_2$). It has been observed that knock-on damage on 2D materials is commonly associated with high acceleration voltage. However, our findings suggest that such damage is more likely to occur at low-Z atoms like Sulfur, while high-Z metal atoms tend to remain intact within the sheet structure. The experimental setup and the following analysis will be described below.

## Sample preparation and experimental setup

TMDs are highly promising for the semiconductor industry as they possess exceptional energy conversion and performance capabilities, making them ideal contenders for next-generation electronic devices. With this particular 2D material, we can obtain excellent diffraction patterns due to the reduction of multiple scattering effects. This ensures that the quality of the patterns is sufficient and of a high standard. High-quality MoS$_2$ and WS$_2$ were mechanically exfoliated from commercial crystals (HQ Graphene) using polydimethylsiloxane (PDMS) sheets, and the sample thickness was identified using photoluminescence (PL) measurements. The samples were subsequently transferred onto the holey silicon nitride TEM grid (Norcada) in our home-built transfer system utilizing a modified viscoelastic stamp technique^30,31^. This procedure yields suspended atomically thin TMDs for TEM analysis.

**Figure 1:**
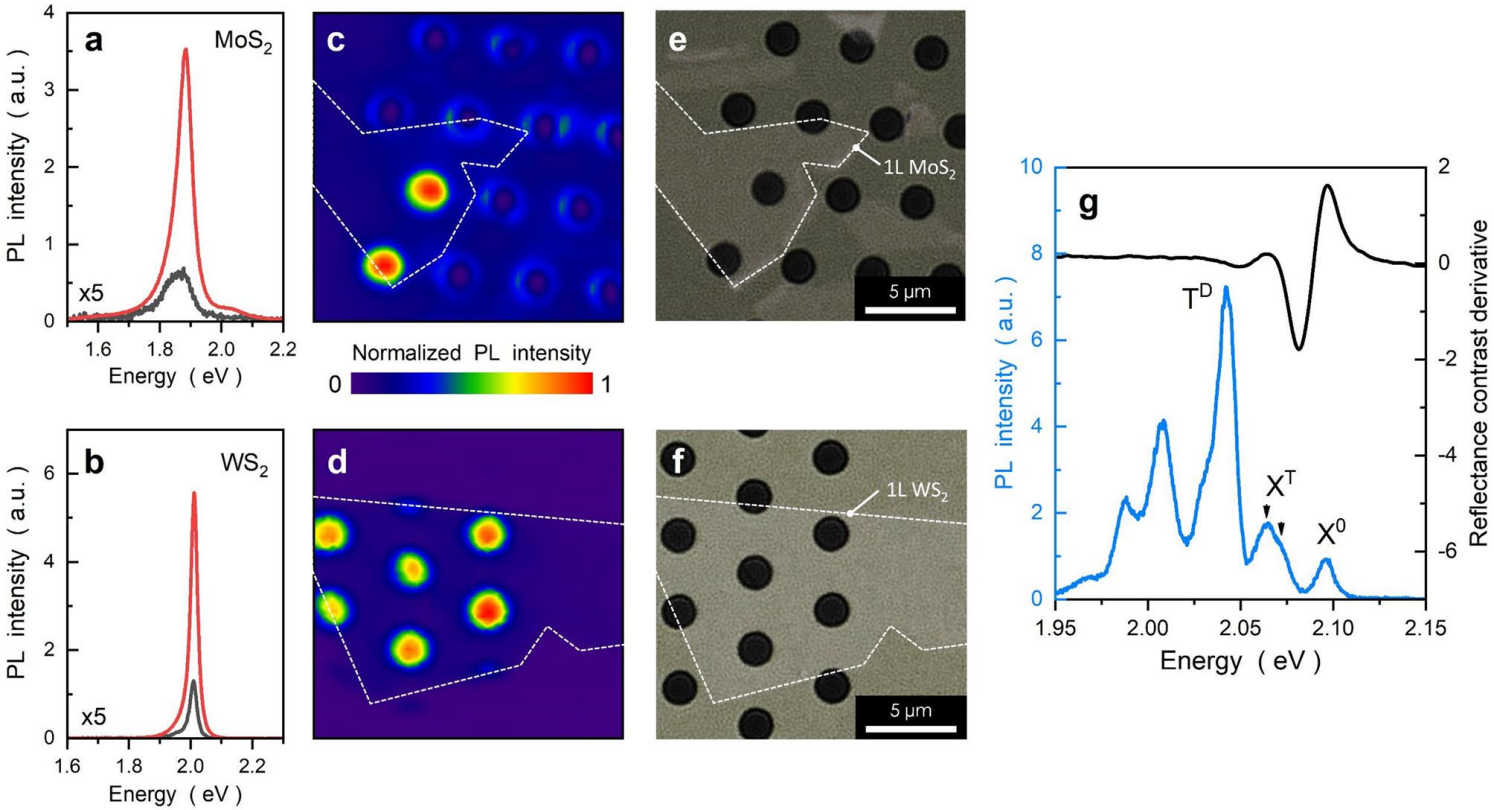
Optical characterization of monolayer MoS$_2$ and WS$_2$. (**a**–**f**) Room-temperature PL spectra (**a**,**b**), PL mappings (**c**,**d**), and optical images (**e**,**f**) of monolayer MoS$_2$ and WS$_2$. Excitons exhibit a strong (weak) PL intensity in the suspended (supported) region, as shown by the red (black) curve. PL spectra obtained from supported regions (black curves) have been zoomed in by a factor of 5. (**g**) PL and derivative reflectance contrast spectra recorded at T= 5K, revealing the neutral exciton (X$^0$) resonance at 2.095 *eV*. Note that multiple emission lines with energies below X$^0$ are typical of exciton complexes in WS$_2$, including trion (X$^T$), dark trion (T$^D$) and their phonon replicas at energies below 2.02 eV. Both singlet- and triplet-spin states of the trion resonance (X$^T$) have been clearly resolved (indicated by arrows). It should be pointed out that the samples are of exceptional quality because: (1) the exciton resonances exhibit a single PL peak with a strong intensity and a narrow linewidth; and (2) the exciton complexes are clearly resolved even without hBN encapsulation. These characteristics reflect the excellent quality and reliability of the 2D samples and fabrication procedures.

All optical measurements were performed in a home-built confocal microscope prior to TEM analysis, as shown in Fig. 1. A continuous-wave 532 nm laser (stable broadband tungsten-halogen lamp) was used for PL (reflectivity) experiments, and the signal was analyzed by a spectrometer equipped with a liquid nitrogen cooled charge-coupled device (CCD). For room-temperature measurements, the excitation sources were focused by a 100$\times$ objective lens ($N.A.= 0.9$), and PL mappings were obtained using a motorized x–y stage with an overall spatial resolution of 0.4 $\mu$m. For low-temperature measurements, the sample was cooled to T = 5K using a closed-cycle cryostat equipped with three-axis nanopositioners, and optical signals were collected using a 30$\times$ objective lens ($N.A. = 0.35$).

The pixelated detector features high frames per second (fps) rate so that we can significantly shorten data acquisition time to a comparable timescale to STEM-HAADF (High-Angle Annular Dark-Field) imaging. The size of the recorded diffraction pattern can extend to 512$\,\times\,$512 pixels (75 $\upmu \text{m}$/pixel) and allow flexible window cropping/binning to 256$\,\times\,$256 or 128$\,\times\,$128 pixels with a maximum speed of up to 16,000 fps. It helps to reduce the effects of drifting that can be caused by unstable stages or thermal vibrations of samples, as well as minimizing radiation damage that can result from extended data collection periods. In terms of dynamic range, the pixelated detector can maintain linearity with a pixel current under 0.2 pA/pixel at an acceleration voltage of 200 *kV* in high-speed mode. Accordingly, the high dynamic range of the diffraction pattern enables the recording of both the intense direct beam in low-frequency regions and diffraction signals in high-frequency regions, while also maintaining a sufficient signal-to-noise ratio (S/N). This ensures accurate results in a single diffraction pattern.

**Figure 2:**
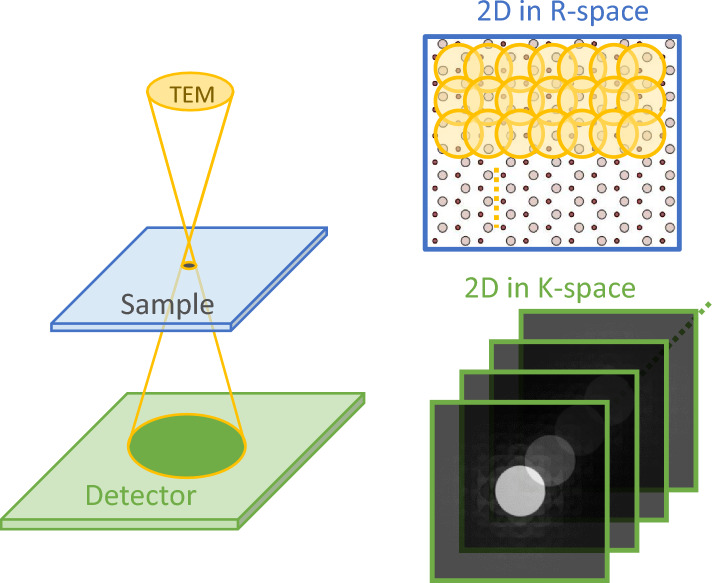
The schematic diagram of 4D STEM. When the electron beam is focused on the specimen, it scans sequentially and involves two spatial coordinates (*X* and *Y*) in real space. At each position, a diffraction pattern of reciprocal space, including two reciprocal coordinates ($k_x$ and $k_y$,) is recorded on the downstream detector. These recordings result in a 4D data cube after integration.

**Figure 3:**
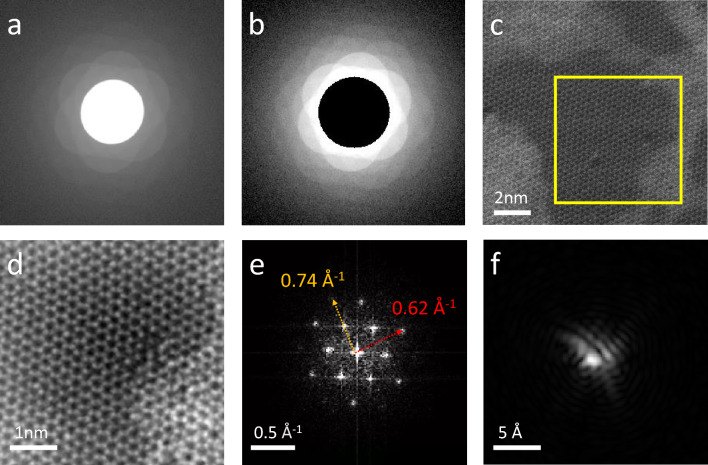
The 4D data acquisition and the reconstruction result of MoS$_2$. (**a**) The summation of all diffraction patterns in log-scale and (**b**) Removement of central beam area to demonstrate the dynamical range in the high-frequency area. (**c**) The HAADF image at 7.2*Mx*. The yellow box indicates the ROI we chose to record the 4D-STEM data. (**d**) The reconstructed object, (**e**) the Fourier analysis of the (**d**), and (**f**) the reconstructed probe of the $1^{st}$ state.

The schematic of 4D STEM is presented in Fig. 2. We collected 4D-STEM data using a Thermo Fisher Titan G2 80-200 ChemiSTEM with a Cs probe corrector, equipped with the Gatan STELA pixelated detector, at a camera length of 165 mm. The data was obtained at 200 kV with an alpha angle of 18.9 mrad. Total 128$\,\times\,$128 steps with the interval 0.6 Å in real space while the size of each diffraction pattern is 512$\,\times\,$512 pixels. For each step, the exposure time is set to 1 ms (i.e. 1000 patterns/s), resulting in a total recording time of approximately 16 seconds. While electron ptychography at 1ms per pixel is slower compared to conventional STEM-HAADF imaging (Fig 3c, 32us per pixel, 1024$\,\times\,$1024, totaling 33 seconds), the overall experiment duration is in similar time scale, the electron dose is about $4.48 \times 10^5\, e^{-}/$ Å$^2$.

## Results and discussion

Before conducting reconstruction, we performed data binning from 512$\,\times\,$512 to 256$\,\times\,$256 to enhance the signal-to-noise (S/N) ratio and padded zeros to the pattern size, extending it to 501p to achieve an increased oversampling ratio. Two mixed-stated probes participated during the reconstruction process by the following equation:

1 $$\begin{aligned}&O_r^{\prime }=O_r+\frac{\alpha }{\max \left( \sum _k\left| P_r^{(k)}\right| ^2\right) } \sum _k P_r^{(k) *} \times \left( \psi _r^{(k)}-P_r^{(k)} O_r\right) \\&P_r^{(k)^{\prime }}=P_r^{(k)}+\frac{\beta }{\max \left( \left| O_r\right| ^2\right) } O_r^{*} \times \left( \psi _r^{(k)}-P_r^{(k)} O_r\right) \end{aligned}$$

where $O_r$ is the object function, $P_r^{(k)}$ is the $k^{th}$ probe function (The superscript “*” represents the complex conjugate), and $\psi _r^{(k)}$ is the wave front in real space (as indicated by the subscript). The constants $\alpha$ and $\beta$ are predetermined variables to fine-tune the ratio of the modification in each iteration.

The data acquisition and reconstructed results are presented in Fig. 3. Both Fig. 3a and b show the summation of diffraction patterns. However, in Fig. 3b, we deliberately covered the direct beam to highlight the exceptional dynamic range of the pixelated detector. It is able to collect intense direct beams while also preserving detailed information in the high-frequency region. As a result, it can effectively record diffraction information of various degrees more completely and linearly. With undistorted high-frequency information, it is possible to accurately retrieve the phase and obtain high-resolution details in real space. The region of interest (ROI) is highlighted in a yellow box in Fig. 3c, which is magnified up to 7.2 *Mx*. The reconstructed object is shown in Fig. 3d, revealing improved visibility of atomic features and intermediate defects compared to Fig. 3b. Fourier analysis was applied to obtain the reciprocal space of the reconstructed object, as depicted in Fig. 3e. We determined that the farthest peak was at (220), based on the Bragg peak, which corresponds to a length of 0.74 Å$^{-1}$. This suggests that our reconstructed resolution is approximately 1.35 Å. As a result, the first state of the mixed-state probes is shown in Fig. 3f.

**Figure 4:**
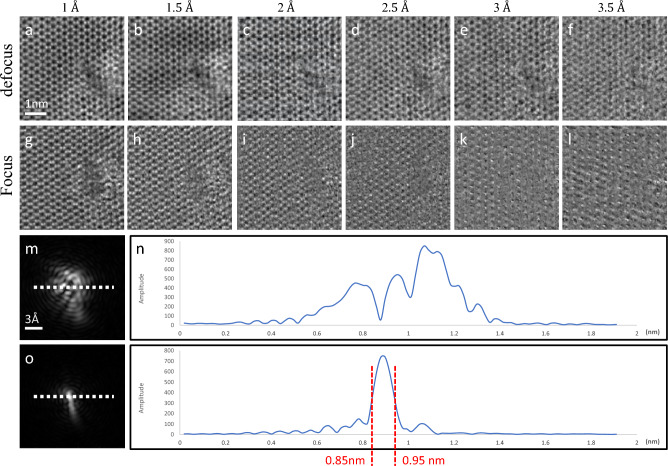
The examination of the step-interval effect on the reconstructed WS$_2$ images. (**a**–**f**) The reconstructed objects at the 20 nm-defocus condition and the step intervals of 1 Å, 1.5 Å, 2 Å, 2.5 Å, 3 Å, and 3.5 Å, respectively. (**g**–**l**) The reconstructed objects at the focus condition and the step intervals of 1 Å, 1.5 Å, 2 Å, 2.5 Å, 3 Å, and 3.5 Å, respectively. (**m**) The reconstructed probe of the 20*nm*-defocus condition and (**n**) the white line profile in (**m**), while (**o**) the reconstructed probe of the focus condition and (**p**) the white line profile in (**o**), indicating a full width half maximum about 1 Å.

To determine the optimal amount of diffraction patterns needed and reduce potential radiation damage during their capture, we conducted reconstructions under focus and defocus conditions using varying step intervals. In Fig. 4, we used a 200 kV acceleration voltage and an alpha angle of 18.9 mrad to gather a series of 4D data with step intervals ranging from 1Å to 3.5Å, at an increment of 0.5 Å. This resulted in a total of 128$\,\times$128 steps in real space and 256$\,\times\,$256 pixels in reciprocal space, producing a 128$\,\times\,$128$\,\times\,$256$\,\times\,$256 diffraction data cube on a WS$_2$ specimen. In the same field of view, we selected 51$\,\times\,$51, 34$\,\times\,$34, 26$\,\times\,$26, 21$\,\times\,$21, 17$\,\times\,$17, and 15$\,\times\,$15 out of 128$\,\times\,$128 diffraction patterns corresponding to step intervals of 1 Å, 1.5 Å, 2 Å, 2.5 Å, 3 Å, and 3.5 Å, respectively.

Based on our predictions, when the size of the probe is reduced to approximately 1 Å, which is known as the focus condition, it leads to a decrease in the overlapping ratio between adjacent incidences when larger step intervals are used. This ultimately causes a decline in the quality of the reconstructed object. Alternatively, the defocus condition produces a much larger probe, resulting in a sufficient overlapping ratio even with increased step intervals. However, as the step interval increases to 3.5 Å, the reconstructed image becomes blurry (as shown in Fig.4f). Our findings suggest that by optimizing the probe size through defocusing, the pixelated detector can maintain a high quality of electron ptychography reconstruction, even when there is a decreased requirement for diffraction patterns (i.e. a reduction in electron dose). The results indicate that under defocused conditions, a step size of 3 Å is adequate for a preliminary atomic shape identification, using an electron dose of roughly $1.27\times 10^4\,e^{-}/$ Å$^2$. Conversely, under focused conditions, a step size of about 1.5 Å provides a similar level of reconstruction quality with an electron dose of $5.08 \times 10^4\, e^{-}/$ Å$^2$.

**Figure 5:**
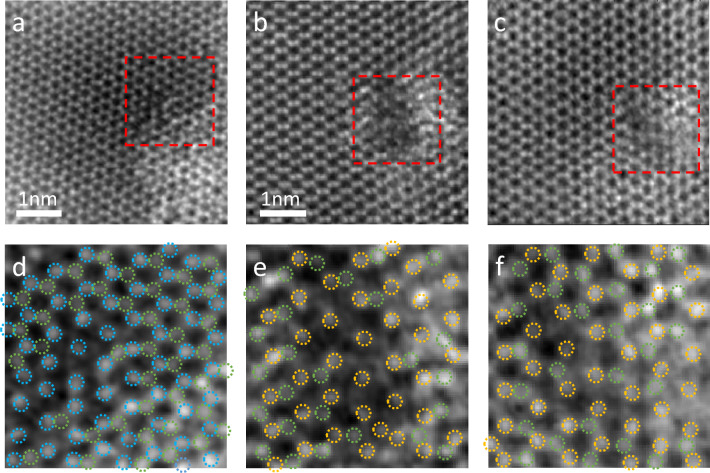
The examination of radiation damage atom-by-atom. (**a**) The reconstructed object of MoS$_2$ at the focus condition. (**b**) The reconstructed WS$_2$ at the focus condition and (**c**) reconstructed WS$_2$ at the defocus condition. (**d**–**f**) correspond to the zoomed views of the red boxes in (**a**–**c**). The blue circles indicate molybdenum atoms, the yellow circles represent tungsten atoms, and the green circles label the sulfur atoms. It is noticeable that there is a region of missing sulfur atoms in the middle regions of all three images, leaving only the high-z atoms (molybdenum or tungsten).

During the experiment, the testing area was exposed to intense radiation, which resulted in defects as seen in Fig. 5. Void formations were observed in both MoS$_2$ (Fig. 5a) and WS$_2$ (Fig. 5b and c) samples due to the electron source’s high acceleration voltage. Interestingly, low-Z atoms like sulfur were displaced from the samples, while high-Z metal atoms such as molybdenum and tungsten were preserved. This suggests that radiation damage is more likely to occur in low-Z atoms than in high-Z metal atoms in the sample. It is also demonstrated that our technique has the capability to detect single-atom defects in the sample. There are some studies have delved extensively into the implications of electron microscopy radiation damage on two-dimensional materials, including predictions regarding radiation dosage limits^32–34^. Moreover, investigations^35,36^ indicate that prolonged electron irradiation of monolayer MoS$_2$ gives rise to the agglomeration of sulfur vacancies into line defects due to the migration of the defects. These line defects consist of single vacancies (SV) and double vacancies (DV) , which are consistent with our results.

## Conclusion

We present the capabilities of the novel 4D-STEM the pixelated detector, STELA, and demonstrate its performance on monolayer 2D materials for electron ptychography. By using high frame rates, the acquisition time is reduced and the results are comparable to conventional STEM-HAADF imaging. Furthermore, the high dynamical range of the pixelated detector collection capacity ensures that scattered electron information is perfectly preserved, which enhances the S/N ratio of diffraction patterns. Moreover, optimizing the defocus can lead to a decrease in electron dose. Finally, although current research on radiation damage in two-dimensional materials has conventionally recommended the adoption of cryo- and low-kV electron microscopy, we contend that our scheme’s high-resolution capabilities can also significantly contribute to the advancement of relevant research. This technology is adept at defect detection and proves to be particularly suitable for exploring local imperfections, such as dislocations or vacancies, within samples.

## Data Availability

The datasets used and analyzed during the current study are available from the corresponding author on reasonable request.

## Code availability

All of our image reconstruction and data analysis source codes will be freely available immediately after this work is published.
